# Exercise-based and mind-body movement interventions for depressive symptoms in patients with inflammatory bowel disease: a systematic review and meta-analysis of randomized controlled trials

**DOI:** 10.3389/fpsyt.2026.1887701

**Published:** 2026-07-29

**Authors:** Zheng Zhang, Juan Li, Li Li, Ling-Feng Ruan

**Affiliations:** 1 Department of Sports Science, Yancheng Institute Of Technology, Yancheng, China; 2 Department of Sports Science, Hanyang University, AnSan, Republic of Korea; 3 Compulsory Treatment Section, The Fourth People’s Hospital of Yancheng, Yancheng, China

**Keywords:** depressive symptoms, exercise, inflammatory bowel disease, meta-analysis, ulcerative colitis

## Abstract

**Background:**

Depressive symptoms are common in patients with inflammatory bowel disease (IBD) and may adversely affect quality of life, treatment adherence, and disease management. Exercise-based and mind–body movement interventions have been proposed as safe and accessible non-pharmacological strategies for improving mental health. However, their effects on depressive symptoms in patients with IBD remain insufficiently synthesized.

**Methods:**

A systematic search was conducted in Embase, the Cochrane Library, PubMed, and Web of Science from inception to May 15 2026. Randomized controlled trials evaluating exercise-based or mind–body movement interventions for depressive symptoms in patients with IBD were included. Two reviewers independently conducted study screening, data extraction, and risk-of-bias assessment. Standardized mean differences (SMDs) with 95% confidence intervals (CIs) were calculated using Review Manager software. Subgroup analyses were performed according to delivery format, intervention modality, intervention duration, and disease type.

**Results:**

A total of 677 records were identified, and 7 articles comprising 9 intervention comparisons were included in the meta-analysis. Baseline depressive symptom scores were comparable between intervention and control groups (SMD , 0.02, 95% CI: −0.38 to 0.42). Compared with control conditions, exercise-based and mind–body movement interventions significantly reduced depressive symptoms in patients with IBD (SMD , −0.49, 95% CI: −0.68 to −0.30, P < 0.00001), with no observed heterogeneity (I² , 0%). Subgroup analyses showed beneficial effects across delivery formats, intervention modalities, intervention durations, and disease types. However, tests for subgroup differences were not statistically significant, and these findings should therefore be interpreted as exploratory trends rather than evidence of superiority of any specific subgroup.

**Conclusion:**

Exercise-based and mind–body movement interventions may reduce depressive symptoms in patients with IBD. The overall effect was consistent across the included studies, but current evidence remains limited by the small number of trials and methodological constraints. Further well-designed randomized controlled trials are needed to clarify the optimal exercise modality, dose, and delivery format for this population.

## 1 Background

Depression is a major contributor to global disease burden and is closely associated with impaired quality of life, reduced social functioning, increased healthcare use, and poorer long-term health outcomes (1, 2). Depressive symptoms are not only common in psychiatric populations but also frequently occur in individuals with chronic physical diseases (3, 4). In these populations, depressive symptoms may amplify symptom perception, reduce adherence to treatment, worsen fatigue and sleep disturbance, and contribute to a cycle of physical inactivity and psychological distress (5, 6). Therefore, identifying safe and accessible strategies to reduce depressive symptoms in patients with chronic disease has become an important public health and clinical priority.

Inflammatory bowel disease (IBD), mainly including Crohn’s disease and ulcerative colitis, is a chronic relapsing inflammatory disorder of the gastrointestinal tract (7). Beyond intestinal symptoms, patients with IBD often experience fatigue, abdominal pain, sleep disturbance, reduced physical functioning, dietary restrictions, and uncertainty related to disease relapse (8–10). These disease-related burdens may increase vulnerability to depressive symptoms. At the same time, depressive symptoms may negatively affect disease self-management, treatment adherence, health-related quality of life, and possibly inflammatory activity through psychoneuroimmune pathways (11, 12). The relationship between IBD and depressive symptoms is therefore likely to be bidirectional, involving biological, psychological, and behavioural mechanisms. Given the chronic and recurrent nature of IBD, effective non-pharmacological approaches for improving mental health in this population are needed.

Exercise has been increasingly recognized as a promising intervention for depressive symptoms. Regular physical activity may reduce depressive symptoms through multiple pathways, including modulation of systemic inflammation, regulation of the hypothalamic–pituitary–adrenal axis, improvement of autonomic function, enhancement of neuroplasticity, and increased release of neurotrophic factors (13, 14). Exercise may also improve sleep, fatigue, self-efficacy, physical function, and social participation, which are particularly relevant for patients with chronic inflammatory conditions (15, 16). In addition to conventional exercise training, mind–body movement interventions such as yoga, Tai Chi, and Qigong may provide additional benefits through gentle movement, breathing regulation, attentional control, and relaxation.

Although several randomized controlled trials have examined exercise-based or mind–body movement interventions in patients with IBD, their effects on depressive symptoms remain insufficiently synthesized (17, 18). Previous evidence has tended to focus on specific modalities, such as yoga, leaving uncertainty regarding whether broader forms of exercise and mind–body movement interventions are associated with improvements in depressive symptoms in this population. Moreover, it remains unclear whether intervention effects differ according to delivery format, intervention modality, duration, or disease type. Therefore, this systematic review and meta-analysis aimed to synthesize randomized controlled trial evidence on the effects of exercise-based and mind–body movement interventions on depressive symptoms in patients with IBD and to explore whether these effects varied across key clinical and intervention characteristics.

## 2 Meta-analysis data

This meta-analysis was performed according to the Preferred Reporting Items for Systematic Reviews and Meta-Analysis statement and the Cochrane Collaboration Handbook. The protocol was registered on PROSPERO (CRD420261395332).

### 2.1 Data sources and searches

A systematic literature search was conducted in four electronic databases: Embase, the Cochrane Library, PubMed, and Web of Science. The databases were searched from inception to May 15, 2026, to identify randomized controlled trials evaluating the effects of exercise-based or mind–body movement interventions on depressive symptoms in patients with inflammatory bowel disease.

The search strategy combined controlled vocabulary terms and free-text keywords related to inflammatory bowel disease, exercise-based interventions, mind–body movement interventions, depressive symptoms, and randomized controlled trials. No restrictions were imposed on publication year. Only studies published in English were included. The detailed search strategies for each database, including the complete search terms and syntax, are provided in **Supplementary File 1**.

Two reviewers (ZZ and LJ) independently conducted the literature search and screened all retrieved records. After duplicate removal, titles and abstracts were assessed for potential eligibility, followed by full-text review according to the predefined inclusion and exclusion criteria. Disagreements were resolved through discussion, and a third reviewer (LL) was consulted when consensus could not be reached. In addition, the reference lists of included studies and relevant reviews were manually checked to identify any additional eligible studies.

### 2.2 Inclusion and exclusion

Studies were eligible for inclusion if they met the following criteria: (1) participants were patients diagnosed with inflammatory bowel disease, including Crohn’s disease, ulcerative colitis, or inflammatory bowel disease not otherwise specified; (2) the intervention consisted of exercise-based or mind–body movement programmes, such as aerobic exercise, resistance training, high-intensity interval training, moderate-intensity continuous training, combined exercise, Tai Chi, Qigong, or yoga; (3) the comparator was usual care, wait-list control, no intervention, health education, or other non-exercise control conditions; (4) depressive symptoms were assessed using validated self-report or clinician-rated scales; and (5) the study was designed as a randomized controlled trial.

Studies were excluded if they met any of the following criteria: (1) participants were not patients with inflammatory bowel disease; (2) the intervention did not include a structured exercise-based or mind–body movement component; (3) depressive symptoms were not reported as an outcome; (4) the study was not a randomized controlled trial, including observational studies, reviews, protocols, case reports, conference abstracts without sufficient data, and animal studies; or (5) sufficient data for effect-size calculation could not be obtained from the article or from the corresponding authors.

Two reviewers (ZZ and LJ) independently assessed study eligibility according to the predefined inclusion and exclusion criteria. Disagreements were resolved through discussion, with consultation from a third reviewer (LL) when necessary.

### 2.3 Assessment of risks of bias

The methodological quality of the included randomized controlled trials was evaluated using the original Cochrane risk-of-bias tool. This tool was used because it is applicable to randomized controlled trials and allows domain-level assessment of key methodological limitations across the included exercise-based and mind–body movement intervention studies. The following domains were assessed: random sequence generation, allocation concealment, blinding of participants and personnel, blinding of outcome assessment, incomplete outcome data, selective reporting, and other potential sources of bias. Each domain was rated as low, unclear, or high risk of bias according to the criteria recommended in the Cochrane Handbook.

In addition to domain-level judgements, an overall study-level risk-of-bias judgement was made for each included trial. A study was considered to have low overall risk of bias when all key domains were rated as low risk, unclear overall risk of bias when at least one key domain was rated as unclear and no key domain was rated as high risk, and high overall risk of bias when at least one key domain was rated as high risk. Risk-of-bias judgements were considered when interpreting the pooled findings and were incorporated into the GRADE assessment of the certainty of evidence.

Risk-of-bias assessment was performed independently by two reviewers (ZZ and LJ). Disagreements were resolved through discussion, with arbitration by a third reviewer (LL) when required. The detailed judgements for each included study are presented in **Supplementary File 2**.

### 2.4 Data extraction

Data were extracted independently by two reviewers (ZZ and LJ) using a predefined data extraction form. Extracted information included the first author, publication year, study design, sample size, participant characteristics, type of inflammatory bowel disease, intervention modality, intervention duration, intervention frequency, session length, comparator condition, depressive symptom outcome measures, follow-up time points, and data required for effect-size calculation.

For continuous outcomes, means, standard deviations, and sample sizes were extracted for both intervention and control groups. When studies reported change scores, post-intervention scores, standard errors, confidence intervals, or other convertible statistics, these data were extracted and transformed where appropriate according to standard meta-analytic methods. If multiple depressive symptom scales or time points were reported, data from the primary depressive symptom outcome and the immediate post-intervention assessment were preferentially extracted.

Any discrepancies in data extraction were resolved through discussion. When consensus could not be reached, a third reviewer (LL) was consulted. If essential data were incomplete or unclear, attempts were made to contact the corresponding authors for additional information.

### 2.5 Assessment of overall effect size

Meta-analyses were performed using Review Manager software (RevMan, version 5.3; The Cochrane Collaboration). Because depressive symptoms were assessed using different validated scales across the included studies, standardized mean differences (SMDs) with 95% confidence intervals (CIs) were calculated as the summary effect measure for continuous outcomes.

For each study, post-intervention depressive symptom scores were extracted for the intervention and control groups. Post-intervention scores were used for the primary meta-analysis because change scores and the corresponding standard deviations of change were not consistently reported across the included trials. Given the randomized controlled design of the included studies and the generally comparable baseline depressive symptom scores between intervention and control groups, pooling post-intervention scores was considered appropriate. When necessary, data were converted into a suitable format for meta-analysis according to standard statistical procedures. A negative SMD indicated lower post-intervention depressive symptom scores in the intervention group compared with the control group.

For multi-arm trials that contributed more than one eligible comparison, the shared control group was divided equally across the relevant comparisons to avoid double-counting participants. The control group mean and standard deviation were kept unchanged, while the control group sample size was split across comparisons. The labels “(1)” and “(2)” indicate distinct intervention comparisons extracted from the same multi-arm trial rather than separate publications.

Statistical heterogeneity was assessed using the chi-square test and the I² statistic. I² values of 25%, 50%, and 75% were considered to represent low, moderate, and high heterogeneity, respectively. A fixed-effect model was used when heterogeneity was low; otherwise, a random-effects model was applied. The pooled effect was considered statistically significant when the 95% CI did not cross zero and the corresponding P value was <0.05.

Sensitivity analysis was performed using a leave-one-out approach, in which each study was sequentially removed from the meta-analysis to examine whether the pooled effect and heterogeneity were substantially influenced by any single study. Because of the limited number of included comparisons, additional sensitivity analyses based on risk of bias, sample size, or intervention subtype were interpreted cautiously.

### 2.6 Subgroup analysis of exercise intervention programmes

Prespecified subgroup analyses were performed to examine whether the pooled effects varied across key intervention and clinical characteristics. Four subgroup variables were considered: delivery format, disease type, intervention modality, and intervention duration. Delivery format was categorized as individual-based or group-based intervention. Disease type was classified as ulcerative colitis, Crohn’s disease, or mixed/unspecified inflammatory bowel disease. Intervention modality was grouped as exercise training, mind–body movement, or combined exercise. Exercise training included resistance training, high-intensity interval training, and moderate-intensity continuous training; mind–body movement included Tai Chi, Qigong, and yoga; and combined exercise referred to multicomponent programmes involving more than one exercise modality. Intervention duration was categorized as ≤12 weeks or >12 weeks.

Subgroup analyses were conducted in Review Manager software (RevMan, version 5.3), and between-subgroup differences were assessed using the RevMan test for subgroup differences. Because the number of included trials was limited and overall heterogeneity was low, these subgroup analyses were considered exploratory and interpreted with caution.

## 3 Result

### 3.1 Search process

**Figure 1:**
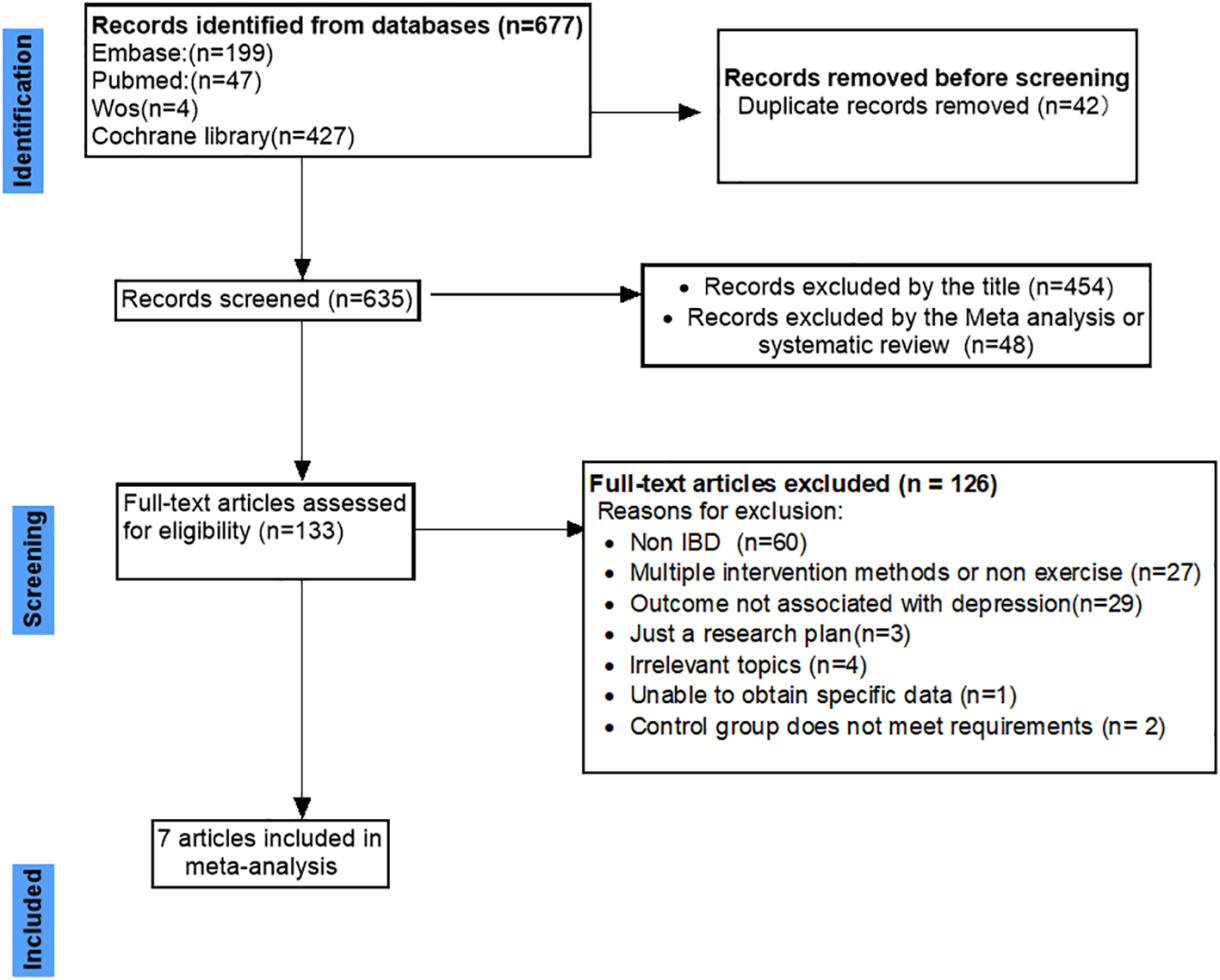
Flowchart and selection of studies.

The study selection process is shown in **Figure 1**. The database search identified 677 records, including 199 records from Embase, 47 from PubMed, 4 from Web of Science, and 427 from the Cochrane Library. After removing 42 duplicates, 635 records were retained for title and abstract screening.

During the screening stage, 454 records were excluded on the basis of title, and 48 records were excluded because they were meta-analyses or systematic reviews. A total of 133 full-text articles were then assessed for eligibility. Of these, 126 articles were excluded for the following reasons: non-IBD populations (n , 60), multiple intervention methods or non-exercise interventions (n , 27), outcomes not related to depression (n , 29), research protocols only (n , 3), irrelevant topics (n , 4), unavailable specific data (n , 1), and ineligible control groups (n , 2). Finally, 7 articles met the inclusion criteria and were included in the meta-analysis.

### 3.2 Characteristics of the included studies and participants

The characteristics of the 7 studies included in this meta-analysis are summarised in **Table 1**. Detailed information is provided on sample size, mean participant age, intervention and control group design, type of exercise intervention, intervention duration and frequency, as well as the depression outcome measures used in each study. Baseline depressive symptom scores and severity levels are presented in **Supplementary File 3**. Across the included comparisons, baseline depressive symptom scores were generally comparable between the intervention and control groups. Most studies used the HADS-D to assess depressive symptoms, while one study used the BDI. Based on commonly used cut-off criteria for these scales, the reported baseline scores generally fell within the non-clinical or minimal range, indicating that most participants did not have clinically relevant depressive symptoms at baseline.

**Table 1:** Characteristics of the included studies and participants.

Studies	Sample size (IG/CG)	Age range (IG/CG)	IG type	Frequency/duration	Disease type
Zhou et al., 2025.(1) (19)	25:24	44.92 ± 14.71/ 42.83 ± 12.14	Taiji	3 times weekly/ 12 weeks	ulcerative colitis
Zhou et al., 2025.(2) (19)	26:24	48.85 ± 14.14/ 42.83 ± 12.14	Resistance training	3 times weekly/ 12 weeks	ulcerative colitis
Tew et al., 2019(1) (20)	13:11	37.0 ± 11.1/ 35.0 ± 10.0	HIIT	3 times weekly/ 12 weeks	Crohn’s disease
Tew et al., 2019(2) (20)	12:11	38.5 ± 13.0/35.0 ± 10.0	MICT	3 times weekly/ 12 weeks	Crohn’s disease
Gerbarg et al., 2015 (21)	14:11		Qigong	26 weeks	IBD
Koch et al., 2021 (22)	47:50		Combined exercise	10 times weekly/ 10 weeks	Crohn’s disease
Peerani et al., 2022 (23)	49:52	45.4 ± 14.0/ 39.7 ± 13.8	Combined exercise	12 weeks	IBD
Cramer et al., 2017 (24)	39:38	45.0 ± 13.3/ 46.1 ± 10.4	Yoga	12 times weekly/ 12 weeks	ulcerative colitis
Bauer et al., 2024 (25)	19:18	49.6 ± 13.1/ 46.8 ± 11.4	Combined Exercise	1 times weekly/ 10 weeks	Crohn’s disease

IG, Intervention Group; CG, Control Group; BDI, Beck Depression Inventory; HADS, Hospital Anxiety Depression Scale; HIIT , High-Intensity Interval Training; MICT , Moderate-Intensity Continuous Training; IBD, Inflammatory Bowel Disease.

None of the included studies clearly reported whether participants were in active flare or clinical remission at baseline. Inflammatory markers such as CRP and fecal calprotectin, as well as detailed symptom-based indicators including diarrhea, abdominal pain, hematochezia, joint pain, and fatigue, were also not consistently available.

### 3.3 Risks of bias

The risk-of-bias assessment is summarized in **Supplementary File 2**. Overall, the included studies showed some methodological concerns, mainly because of the difficulty of blinding participants and personnel in exercise-based and mind–body movement interventions. Random sequence generation was generally judged to be at low risk of bias, indicating that most trials reported an appropriate randomization process. Allocation concealment was rated as low risk in several studies but was unclear in some trials because insufficient methodological details were provided (**Table 2**).

**Table 2:** GRADE assessment for the primary outcome.

Outcome	No. of studies	Participants	Effect estimate	Certainty of evidence	Reasons for downgrading
Depressive symptoms	7 RCTs	448	SMD, -0.49, 95% CI: −0.68 to −0.30	Low	Downgraded one level for risk of bias because blinding was difficult and some domains were unclear; downgraded one level for imprecision because of the small number of studies and limited sample size.

Blinding of participants and personnel was the most common source of bias. Most studies were judged to be at high risk in this domain, which is expected in exercise-based and mind–body movement trials where participant blinding is difficult to implement. Blinding of outcome assessment was generally rated as low or unclear risk, although one study was judged to be at high risk. Incomplete outcome data were mostly assessed as low risk, suggesting that attrition was generally well managed. Selective reporting was rated as low risk in several studies but remained unclear in others because of limited protocol or registration information. Other potential sources of bias were mainly judged as low or unclear risk.

Taken together, the included trials had important risk-of-bias considerations, particularly the high risk of performance bias and unclear reporting in some domains. These issues were considered in the GRADE assessment and should be taken into account when interpreting the pooled findings.

### 3.4 Meta-analysis

#### 3.4.1 Baseline comparability

**Figure 2:**
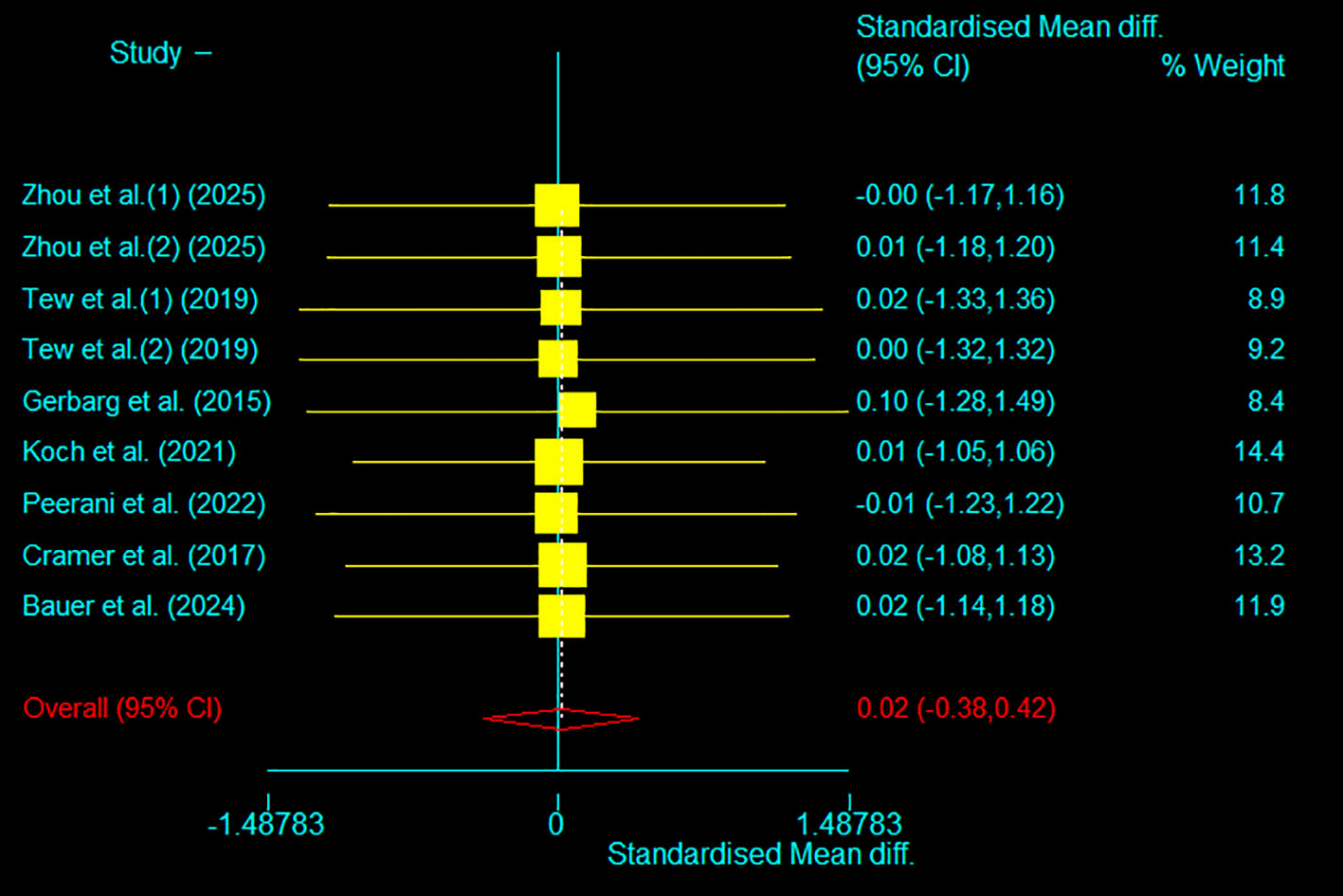
Baseline period test.

Baseline comparability between the intervention and control groups was assessed using pre-intervention depressive symptom scores. The pooled analysis showed no significant difference between groups at baseline (SMD , 0.02, 95% CI: −0.38 to 0.42), indicating that depressive symptom levels were comparable before the intervention. The confidence interval crossed zero, and the overall effect size was close to null, suggesting no evidence of baseline imbalance between groups. The detailed forest plot is shown in **Figure 2**.

#### 3.4.2 Overall effect of exercise interventions on depressive symptoms

The pooled analysis included 9 intervention comparisons from 7 studies, involving 244 participants in the exercise intervention groups and 204 participants in the control groups. Compared with control conditions, exercise-based and mind–body movement interventions significantly reduced depressive symptoms in patients with inflammatory bowel disease (SMD , −0.49, 95% CI: −0.68 to −0.30, P < 0.00001). The negative effect size indicates that the intervention groups showed greater improvements in depressive symptoms than the control groups.

**Figure 3:**
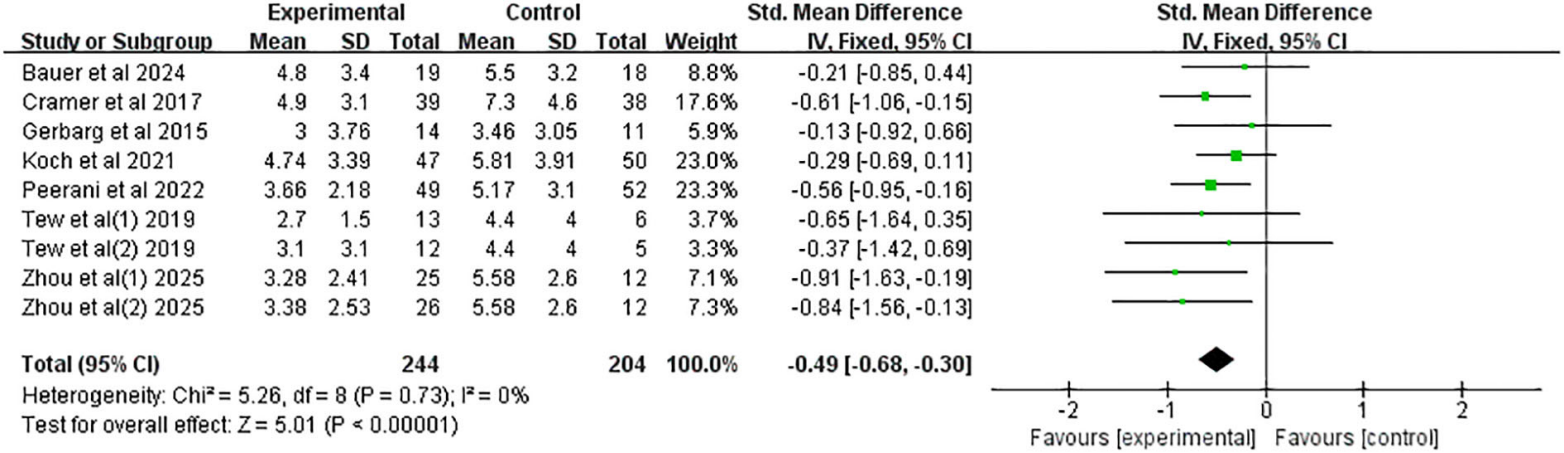
Forest plot of exercise interventions for depressive symptoms in patients with IBD.

No significant between-study heterogeneity was observed (I², 0%), suggesting that the intervention effects were consistent across the included comparisons. The detailed forest plot is shown in **Figure 3**.

#### 3.4.3 Assessment of publication bias

Publication bias was not formally assessed because fewer than ten intervention comparisons were included. Under these conditions, funnel plot asymmetry and statistical tests for small-study effects, such as Egger’s regression test, are generally considered unreliable. Therefore, no definitive conclusion regarding publication bias could be drawn.

#### 3.4.4 Subgroup analysis

Subgroup analyses were conducted to explore whether the effects of exercise-based and mind–body movement interventions on depressive symptoms differed across key clinical and intervention characteristics. Four subgroup variables were examined: delivery format, disease type, intervention modality, and intervention duration. Delivery format was classified as individual-based or group-based intervention. Disease type was categorized as ulcerative colitis, Crohn’s disease, or mixed/unspecified inflammatory bowel disease. Intervention modality was classified as exercise training, mind–body movement, or combined exercise. Intervention duration was categorized according to the predefined duration groups.

Given the low heterogeneity observed in the overall meta-analysis, these subgroup analyses were not intended to explain substantial between-study heterogeneity. Instead, they were performed as exploratory analyses to assess the consistency of intervention effects across different study and intervention characteristics. Therefore, the subgroup findings should be interpreted cautiously, particularly because of the limited number of studies within some subgroups.

##### 3.4.4.1 Delivery format

Subgroup analysis by delivery format showed that both individual-based and group-based interventions were associated with reductions in depressive symptoms. The pooled effect for individual-based interventions was SMD , −0.68 (95% CI: −0.99 to −0.37, P < 0.0001), with no observed heterogeneity (I² , 0%). Group-based interventions also showed a significant reduction in depressive symptoms (SMD , −0.38, 95% CI: −0.62 to −0.13, P , 0.002), again with no observed heterogeneity (I² , 0%).

**Figure 4:**
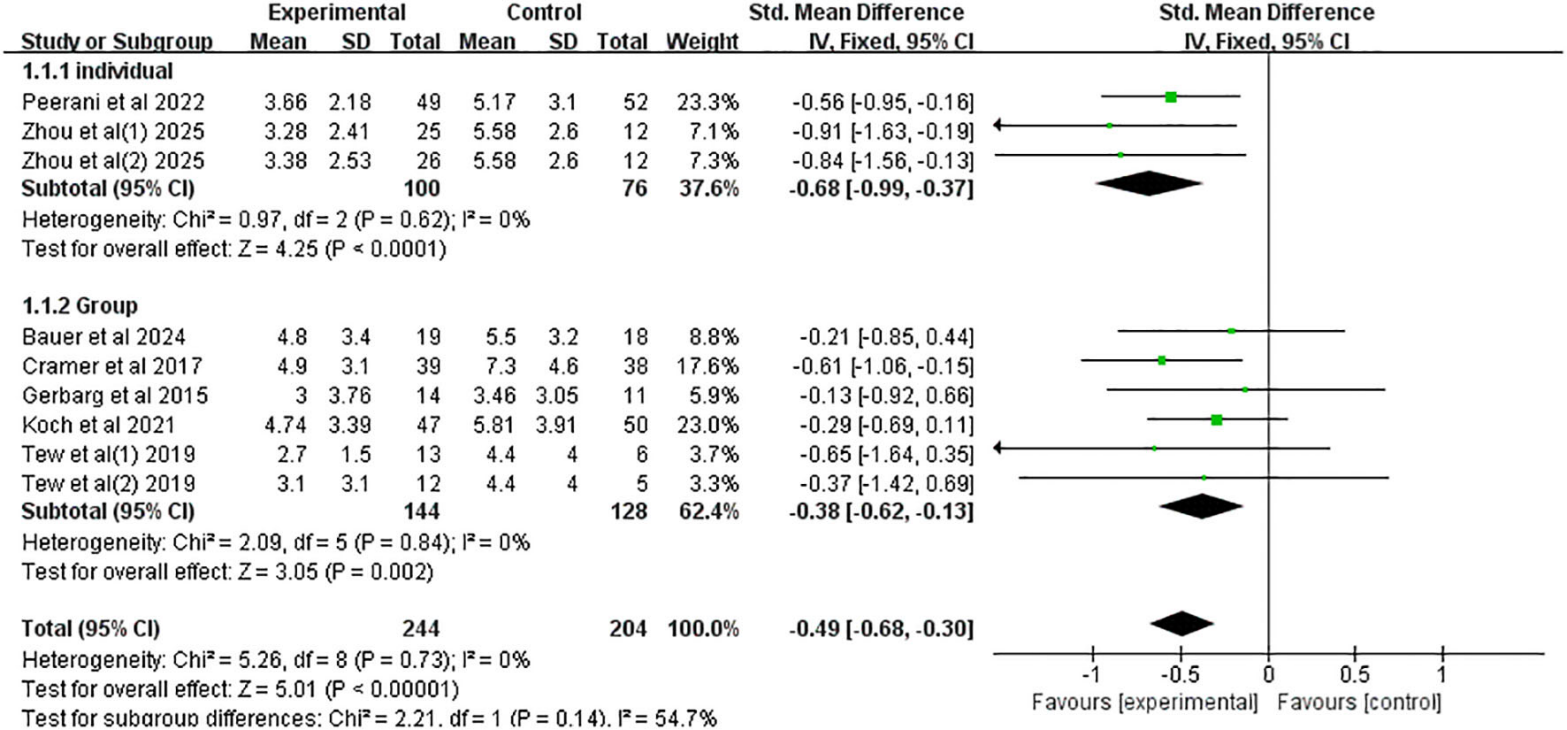
Forest plot of subgroup analyses by intervention format.

The test for subgroup differences did not reach statistical significance (χ² , 2.21, df , 1, P , 0.14), although the effect estimate appeared numerically larger for individual-based interventions than for group-based interventions. This pattern may suggest a potentially stronger trend for individually delivered programmes; however, given the non-significant subgroup difference and the limited number of studies, this finding should be interpreted cautiously. The detailed forest plot is shown in **Figure 4**.

##### 3.4.4.2 Intervention modality

Subgroup analysis by intervention modality showed that depressive symptoms were reduced across all three categories of interventions. Exercise training interventions were associated with a significant reduction in depressive symptoms (SMD , −0.68, 95% CI: −1.19 to −0.17, P , 0.008), with no observed heterogeneity (I² , 0%). Mind–body movement interventions also showed a significant effect (SMD , −0.58, 95% CI: −0.93 to −0.24, P , 0.001), with low heterogeneity (I² , 3%). Combined exercise interventions demonstrated a smaller but statistically significant reduction in depressive symptoms (SMD , −0.39, 95% CI: −0.65 to −0.13, P , 0.003), with no observed heterogeneity (I² , 0%).

**Figure 5:**
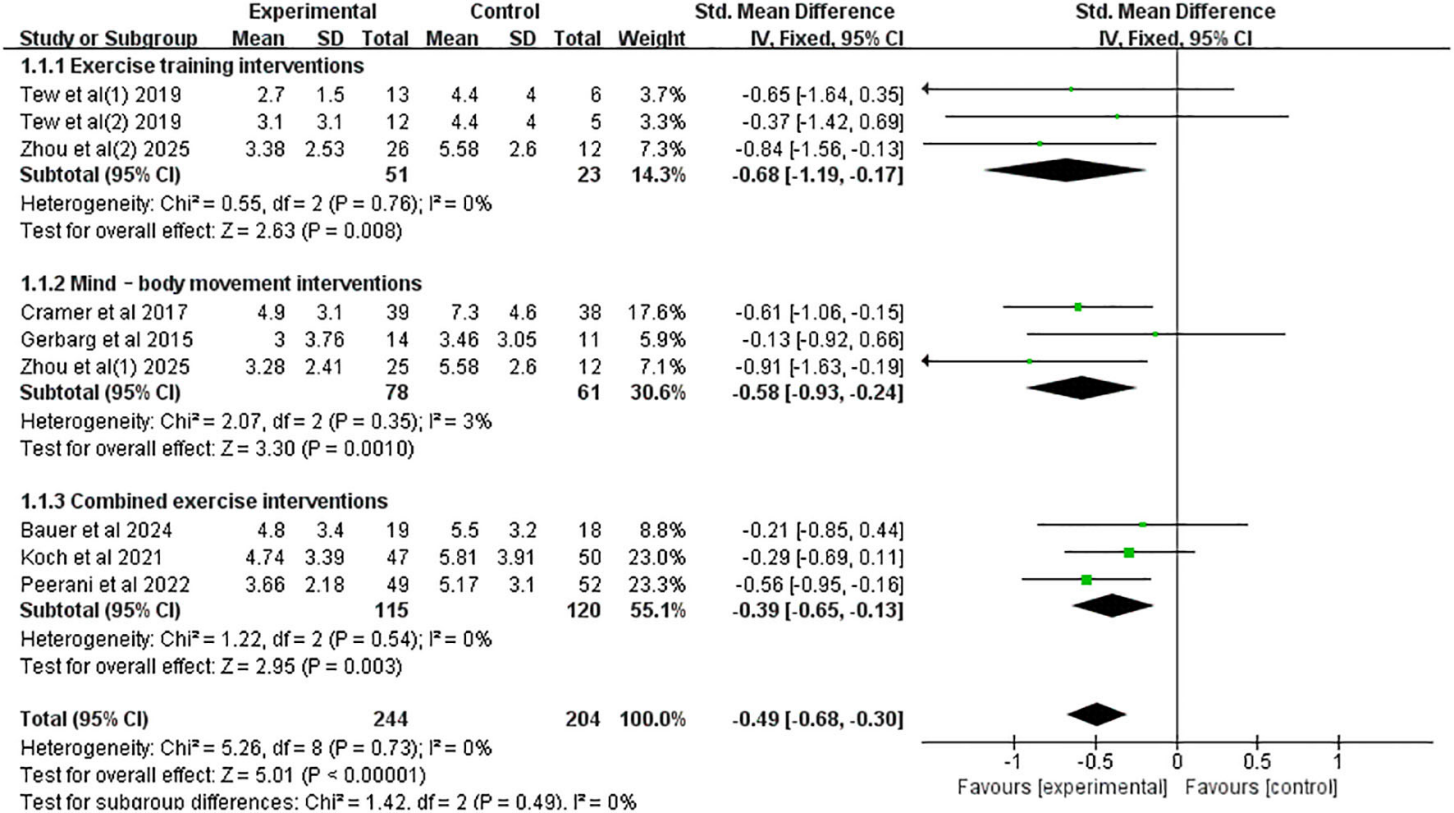
Forest plot of subgroup analyses by exercise modality.

The test for subgroup differences was not statistically significant (χ² , 1.42, df , 2, P , 0.49), indicating no clear evidence that intervention effects differed by modality. Although the pooled estimates appeared numerically larger for exercise training and mind–body movement interventions than for combined exercise interventions, this pattern should be interpreted only as a possible trend rather than evidence of superiority. The detailed forest plot is shown in **Figure 5**.

##### 3.4.4.3 Intervention duration

Subgroup analysis by intervention duration showed that interventions lasting ≤12 weeks were associated with a significant reduction in depressive symptoms (SMD , −0.51, 95% CI: −0.71 to −0.32, P < 0.00001), with no observed heterogeneity (I² , 0%). For interventions lasting >12 weeks, the pooled estimate was smaller and not statistically significant (SMD , −0.13, 95% CI: −0.92 to 0.66, P , 0.75).

**Figure 6:**
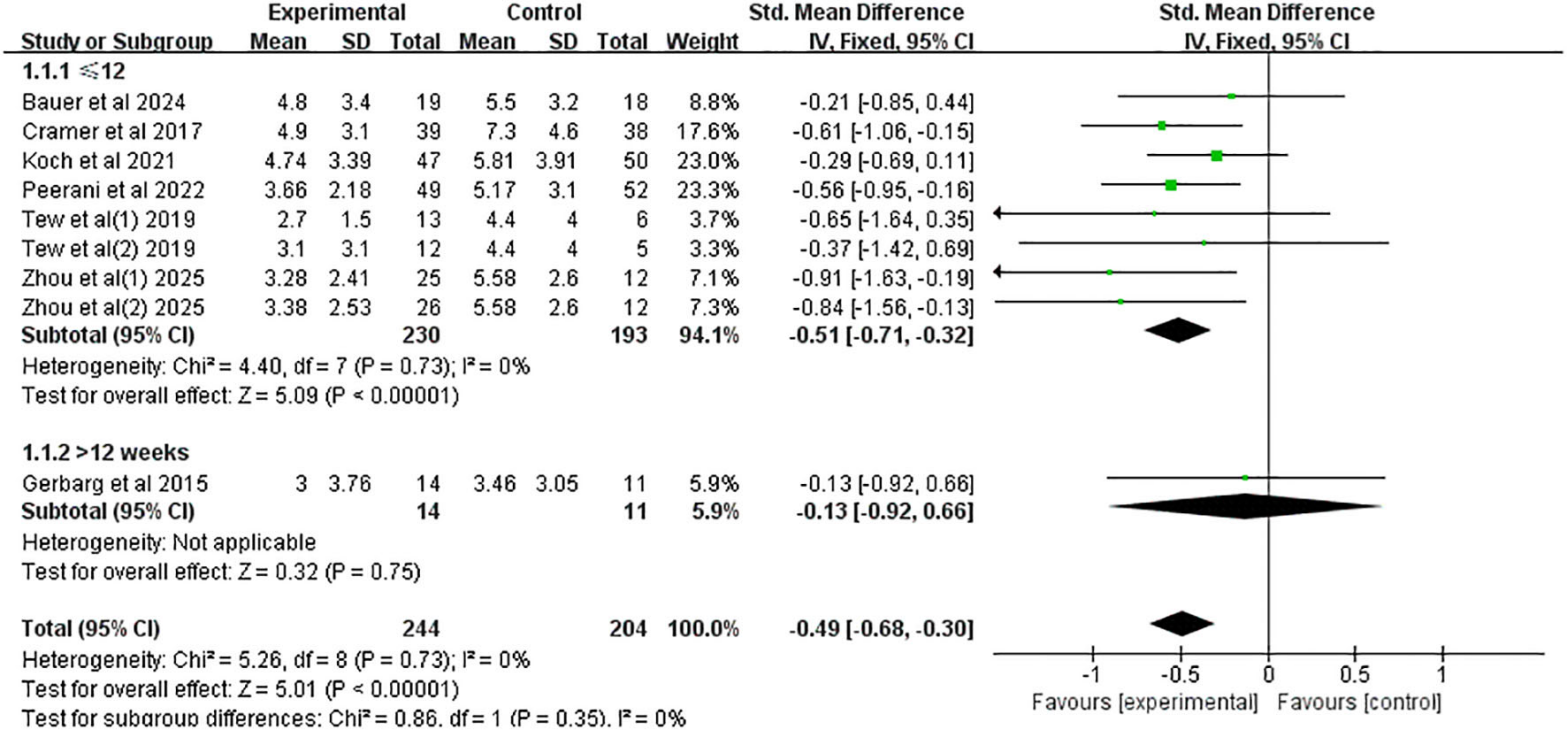
Forest plot of subgroup analyses by intervention duration.

The test for subgroup differences was not statistically significant (χ² , 0.86, df , 1, P , 0.35), indicating no clear evidence that intervention effects differed by duration. Although the effect estimate appeared more pronounced in interventions lasting ≤12 weeks, this should be interpreted only as a possible trend rather than evidence that shorter interventions are superior. The >12-week subgroup included only one comparison, further limiting the robustness of this finding. The detailed forest plot is shown in **Figure 6**.

##### 3.4.4.4 Disease type

Subgroup analysis by disease type showed that exercise-based and mind–body movement interventions were associated with reductions in depressive symptoms across different IBD populations. In studies involving patients with ulcerative colitis, the pooled effect was statistically significant (SMD , −0.73, 95% CI: −1.07 to −0.39, P < 0.0001), with no observed heterogeneity (I² , 0%). A smaller effect was observed in studies involving patients with Crohn’s disease and reached borderline statistical significance (SMD , −0.31, 95% CI: −0.62 to −0.00, P , 0.05), with no observed heterogeneity (I² , 0%). For studies including mixed IBD populations, the pooled effect was also statistically significant (SMD , −0.47, 95% CI: −0.83 to −0.11, P , 0.010), again with no observed heterogeneity (I² , 0%).

**Figure 7:**
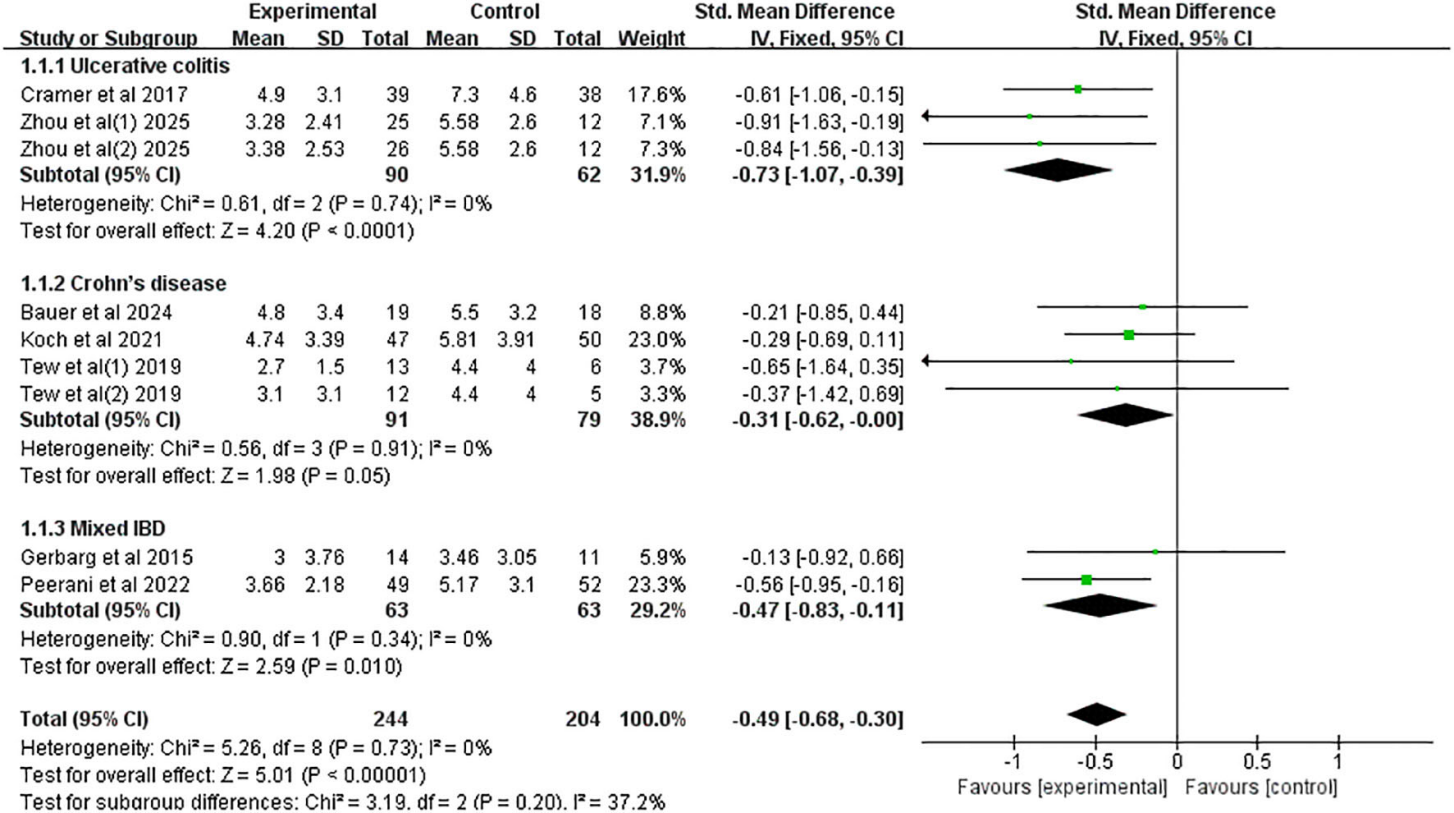
Forest plot of subgroup analyses by disease type.

The test for subgroup differences was not statistically significant (χ² , 3.19, df , 2, P , 0.20), indicating no clear evidence that intervention effects differed by disease type. Although the effect estimate appeared numerically larger in ulcerative colitis studies than in Crohn’s disease or mixed IBD studies, this should be interpreted only as a possible exploratory trend rather than evidence of a stronger effect in this subgroup. The detailed forest plot is shown in **Figure 7**.

### 3.5 Certainty of evidence

Using the GRADE approach, the certainty of evidence for depressive symptoms was rated as low. The evidence was downgraded mainly because of concerns regarding risk of bias and imprecision due to the small number of included trials and limited sample size. Although statistical heterogeneity was negligible, clinical heterogeneity related to intervention modality, disease characteristics, and baseline depressive symptom severity was also considered when interpreting the certainty of evidence. The GRADE Summary of Findings table is provided in **Supplementary Table 2**.

### 3.6 Sensitivity analysis

Leave-one-out sensitivity analysis showed that sequential removal of each individual study did not materially change the direction of the pooled effect. Statistical heterogeneity also remained negligible or low across the analyses, suggesting that the overall finding was not driven by any single study. However, because the number of included comparisons was small, these sensitivity findings should be interpreted cautiously.

## 4 Discussion

This systematic review and meta-analysis synthesized randomized controlled trial evidence on exercise-based and mind–body movement interventions for depressive symptoms in patients with IBD. The pooled results showed that these interventions were associated with a significant reduction in depressive symptoms compared with control conditions. Baseline depressive symptom scores were comparable between intervention and control groups, suggesting that the observed post-intervention effects were unlikely to be explained by baseline imbalance. Although statistical heterogeneity was negligible, this should not be interpreted as evidence of clinical homogeneity. The included interventions differed substantially in content, intensity, duration, delivery format, and presumed physiological and psychological mechanisms. Therefore, the pooled estimate should be interpreted as the average effect across a clinically diverse group of exercise-based and mind–body movement interventions. Subgroup analyses further suggested that the beneficial effects were generally observed across different delivery formats, intervention modalities, intervention durations, and disease types. However, because tests for subgroup differences were not statistically significant and the number of studies within several subgroups was limited, these findings should be interpreted as exploratory trends rather than evidence that one intervention format, modality, duration, or disease subtype was superior to another.

Although the pooled effect was statistically significant, its clinical meaningfulness requires careful interpretation. The effect size of SMD , −0.49 represents a moderate effect according to conventional criteria, indicating that exercise-based and mind–body movement interventions may have a potentially meaningful impact on depressive symptoms in patients with IBD. However, because the included trials used different depressive symptom scales and rarely reported minimal clinically important difference thresholds, it remains unclear whether the observed standardized effect corresponds to a clinically important improvement for individual patients. In addition, as many participants had non-clinical or minimal depressive symptoms at baseline, the clinical impact may differ between general IBD populations and patients with clinically relevant depression. Future studies should evaluate scale-specific changes, minimal clinically important differences, and patient-centered outcomes to better determine the clinical relevance of these interventions.

Our findings extend previous evidence on mind–body exercise for IBD. A previous meta-analysis focusing specifically on yoga reported that yoga-based interventions may improve psychological outcomes, including depressive symptoms, in patients with IBD (26). Compared with that work, the present study adopted a broader intervention scope by including not only yoga, but also Tai Chi, Qigong, resistance training, high-intensity interval training, moderate-intensity continuous training, and combined exercise programmes. This wider inclusion allowed us to evaluate exercise-based and mind–body movement interventions within a more comprehensive framework. In addition, the present study explored whether intervention effects varied according to delivery format, intervention modality, duration, and disease type. Although these subgroup analyses did not show statistically significant between-group differences, they provide preliminary information on possible patterns of response and may help guide the design of future trials.

Several biological and psychosocial mechanisms may explain the observed association between exercise interventions and reduced depressive symptoms in patients with IBD. First, exercise may modulate systemic inflammation, which is relevant because IBD is characterized by chronic immune activation and inflammatory burden. Regular physical activity may reduce pro-inflammatory cytokine activity and improve immune regulation, thereby influencing inflammatory pathways that are also implicated in depression (27). Second, exercise may improve hypothalamic–pituitary–adrenal axis regulation and autonomic balance, which could reduce stress reactivity and improve emotional regulation (28). Third, physical activity may promote neurobiological adaptations, including increased neurotrophic signaling, improved neurotransmitter regulation, and enhanced neuroplasticity, all of which are relevant to depressive symptoms (29). Fourth, exercise and mind–body movement may improve sleep quality, fatigue, physical function, and perceived self-efficacy, which are common concerns in patients with IBD and may indirectly contribute to better mental health (30–32). Mind–body interventions such as yoga, Tai Chi, and Qigong may additionally combine gentle movement, breathing regulation, attentional control, and relaxation, which may help reduce psychological distress through both physiological and cognitive-affective pathways (33).

Baseline depressive symptom severity may have influenced the observed intervention effects. As shown in **Supplementary File 3**, most included comparisons reported baseline depressive symptom scores within the non-clinical or minimal range according to commonly used scale-specific cut-off criteria. This suggests that many participants had relatively low depressive symptom burden at baseline, which may have limited the potential magnitude of improvement and introduced a possible floor effect. Therefore, the pooled effect should be interpreted as the effect of exercise-based and mind–body movement interventions on depressive symptoms in general IBD populations rather than specifically in patients with clinically diagnosed depression. Future trials should consider recruiting patients with clinically relevant depressive symptoms or conducting subgroup analyses according to baseline depressive symptom severity.

This study has several limitations. First, the number of included randomized controlled trials was small, and several subgroup categories contained only a limited number of comparisons. Therefore, the subgroup findings should be regarded as exploratory and hypothesis-generating. Second, although statistical heterogeneity was low, clinical and methodological differences existed across studies, including variations in disease type, intervention modality, intervention duration, delivery format, control conditions, and depressive symptom scales. Third, information on concomitant pharmacological treatment was limited across the included trials. Medication use, particularly biologic therapy, corticosteroids, immunomodulators, and other IBD-related treatments, may influence intestinal inflammation, disease activity, pain, fatigue, and other symptoms, which may in turn affect depressive symptoms. However, the included trials did not consistently report medication use, biologic therapy status, or medication stability during the intervention period. Therefore, the independent contribution of exercise-based and mind–body movement interventions beyond pharmacological disease control could not be fully determined. Fourth, disease activity status was another important clinical factor that could not be fully assessed. The included studies did not clearly report whether participants were experiencing active flare or were in clinical remission at baseline. Inflammatory markers such as CRP and fecal calprotectin, as well as symptom-based indicators including diarrhea, abdominal pain, hematochezia, joint pain, and fatigue, were also insufficiently reported. This limited our ability to determine whether disease activity status influenced depressive symptom outcomes or modified the effects of exercise-based and mind–body movement interventions. Fifth, blinding of participants and personnel was generally difficult to achieve in exercise-based interventions, leading to a high risk of performance bias in several studies. These risk-of-bias concerns reduced confidence in the pooled estimate and were reflected in the low certainty of evidence in the GRADE assessment. Sixth, some risk-of-bias domains were rated as unclear because of incomplete reporting, particularly regarding allocation concealment, selective reporting, or other potential sources of bias. Seventh, publication bias could not be formally assessed because fewer than ten comparisons were available. Finally, most included studies assessed short-term post-intervention outcomes, and evidence remains insufficient regarding the long-term sustainability of exercise-related improvements in depressive symptoms among patients with IBD.

Overall, this meta-analysis suggests that exercise-based and mind–body movement interventions may reduce depressive symptoms in patients with IBD. The effects appeared broadly consistent across different intervention and clinical characteristics, but current subgroup findings should be interpreted cautiously. Larger, well-designed randomized controlled trials with standardized intervention protocols, longer follow-up periods, and improved reporting quality are needed to clarify the optimal exercise modality, delivery format, and dose for improving depressive symptoms in this population.

## 5 Conclusion

This systematic review and meta-analysis suggests that exercise-based and mind–body movement interventions may significantly reduce depressive symptoms in patients with IBD. The overall effect was consistent across the included studies, with low statistical heterogeneity. Exploratory subgroup analyses indicated broadly similar beneficial trends across delivery format, intervention modality, intervention duration, and disease type, although no clear evidence of subgroup differences was observed. Given the limited number of trials and the methodological limitations of some studies, further well-designed randomized controlled trials are needed to determine the optimal exercise modality, dose, and delivery format for this population.

## Data Availability

The original contributions presented in the study are included in the article/**Supplementary Material**. Further inquiries can be directed to the corresponding author.
