# Supplementary material for: Exercise-based and mind-body movement interventions for depressive symptoms in patients with inflammatory bowel disease: a systematic review and meta-analysis of randomized controlled trials
Source: Front Psychiatry. 2026 Jul 29;17:1887701. doi: 10.3389/fpsyt.2026.1887701 (PMC13461715; doi:10.3389/fpsyt.2026.1887701)
Supplement: Supplementary file 1 [file Supplementaryfile2.pdf]

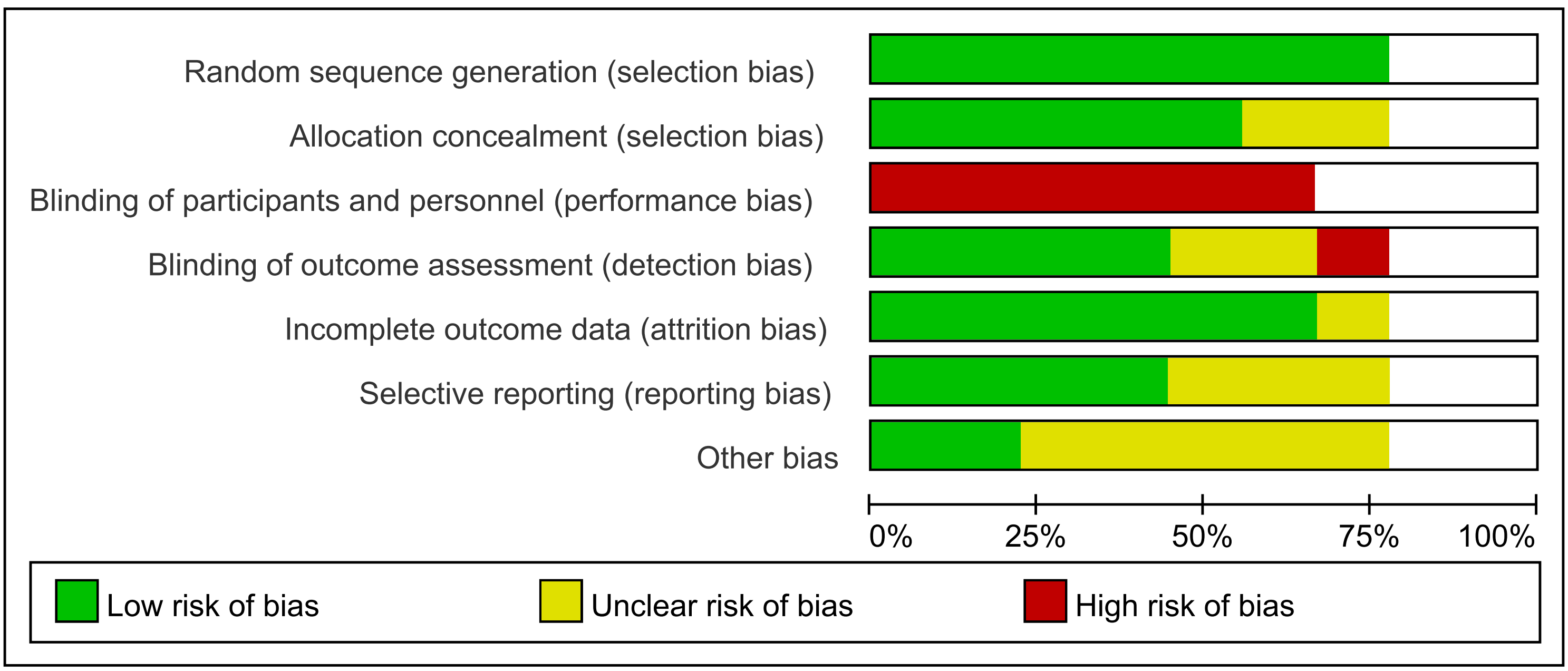
Random sequence generation (selection bias)
Allocation concealment (selection bias)
Blinding of participants and personnel (performance bias)
Blinding of outcome assessment (detection bias)
Incomplete outcome data (attrition bias)
Selective reporting (reporting bias)
Other bias
0%
25%
50%
75%
100%
Low risk of bias
Unclear risk of bias
High risk of bias
