# Supplementary material for: Exercise-based and mind-body movement interventions for depressive symptoms in patients with inflammatory bowel disease: a systematic review and meta-analysis of randomized controlled trials
Source: Front Psychiatry. 2026 Jul 29;17:1887701. doi: 10.3389/fpsyt.2026.1887701 (PMC13461715; doi:10.3389/fpsyt.2026.1887701)
Supplement: Supplementary file 2 [file Supplementaryfile1.docx]

**Pubmed search algorithm**

#1	"Exercise"[Mesh Terms]

#2	"Exercises"[Title/Abstract] OR "physical activity"[Title/Abstract] OR "activities physical"[Title/Abstract] OR "activity physical"[Title/Abstract] OR "physical activities"[Title/Abstract] OR "exercise physical"[Title/Abstract] OR "exercises physical"[Title/Abstract] OR "physical exercise"[Title/Abstract] OR "physical exercises"[Title/Abstract] OR "acute exercise"[Title/Abstract] OR "acute exercises"[Title/Abstract] OR "exercise acute"[Title/Abstract] OR "exercises acute"[Title/Abstract] OR "exercise isometric"[Title/Abstract] OR "exercises isometric"[Title/Abstract] OR "isometric exercises"[Title/Abstract] OR "isometric exercise"[Title/Abstract] OR "exercise aerobic"[Title/Abstract] OR "aerobic exercise"[Title/Abstract] OR "aerobic exercises"[Title/Abstract] OR "exercises aerobic"[Title/Abstract] OR "exercise training"[Title/Abstract] OR "exercise trainings"[Title/Abstract] OR "training exercise"[Title/Abstract] OR (("education"[MeSH Subheading] OR "education"[All Fields] OR "Training"[All Fields] OR "education"[MeSH Terms] OR "train"[All Fields] OR "train s"[All Fields] OR "trained"[All Fields] OR "training s"[All Fields] OR "Trainings"[All Fields] OR "trains"[All Fields]) AND "Exercise"[Title/Abstract])

#3	#1 OR #2

#4	"Inflammatory Bowel Diseases"[Mesh Terms]

#5 "Inflammatory Bowel Disease"[Title/Abstract] OR "Bowel Diseases, Inflammatory"[Title/Abstract] OR "inflammatory bowel disease"[Title/Abstract] OR " Crohn's disease "[Title/Abstract] OR "ulcerative colitis"[Title/Abstract]

#6	#4 OR #5

#7 "randomized controlled trial"[Publication Type] OR "randomized"[Title/Abstract] OR "placebo"[Title/Abstract]

#8 #3 AND #6 AND #7

**Web of Science search algorithm**

#1 TS=(“Exercise, Acute” OR “Exercises, Acute” OR “Exercise, Isometric” OR “Exercises, Isometric” OR “Isometric Exercises” OR “Isometric Exercise” OR “Exercise, Aerobic” OR “Aerobic Exercise” OR “Aerobic Exercises” OR “Exercises, Aerobic” OR “Exercise Training” OR “Exercise Trainings” OR “Training, Exercise” OR “Trainings, Exercise”) and Preprint Citation Index (Exclude – Database)

#2 TS=(“Inflammatory Bowel Diseases” OR “Inflammatory Bowel Disease” OR “inflammatory bowel disease” OR “inflammatory bowel disease” OR “Crohn's disease”OR “ulcerative colitis”) and Preprint Citation Index (Exclude – Database)

#3 TS=(“randomized controlled trial” OR “randomized” OR “placebo” OR “RCT”) and Preprint Citation Index (Exclude – Database)

#4 #1 AND #2 AND #3

**Embase search algorithm**

#1	exercise

#2	‘exercises':ab,ti OR 'physical activity':ab,ti OR 'activities, physical':ab,ti OR 'activity, physical':ab,ti OR 'physical activities':ab,ti OR 'exercise, physical':ab,ti OR 'exercises, physical':ab,ti OR 'physical exercise':ab,ti OR 'physical exercises':ab,ti OR 'acute exercise':ab,ti OR 'acute exercises':ab,ti OR 'exercise, acute':ab,ti OR 'exercises, acute':ab,ti OR 'exercise, isometric':ab,ti OR 'exercises, isometric':ab,ti OR 'isometric exercises':ab,ti OR 'isometric exercise':ab,ti OR 'exercise, aerobic':ab,ti OR 'aerobic exercise':ab,ti OR 'aerobic exercises':ab,ti OR 'exercises, aerobic':ab,ti OR 'exercise training':ab,ti OR 'exercise trainings':ab,ti OR 'training, exercise':ab,ti OR 'trainings, exercise':ab,ti

#3	#1 OR #2

#4	'Inflammatory Bowel Diseases':ab,ti OR 'Inflammatory Bowel Disease':ab,ti OR 'Bowel Diseases, Inflammatory':ab,ti OR 'inflammatory bowel disease':ab,ti OR 'Crohn’s disease':ab,ti OR ' ulcerative colitis ':ab,ti

#5	'randomized controlled trial':ab,ti OR 'randomized':ab,ti OR 'placebo':ab,ti OR 'rct':ab,ti

#6	#3 AND #4 AND #5

**Cochrane Library search algorithm**

#1	exercise

#2	(Exercises):ab,ti,kw OR (Physical Activity):ab,ti,kw OR (Activities, Physical):ab,ti,kw OR (Activity, Physical):ab,ti,kw OR (Physical Activities):ab,ti,kw OR (Exercise, Physical):ab,ti,kw OR (Exercises, Physical):ab,ti,kw OR (Physical Exercise):ab,ti,kw OR (Physical Exercises):ab,ti,kw OR (Acute Exercise):ab,ti,kw OR (Acute Exercises):ab,ti,kw OR (Exercise, Acute):ab,ti,kw OR (Exercises, Acute):ab,ti,kw OR (Exercise, Isometric):ab,ti,kw OR (Exercises, Isometric):ab,ti,kw OR (Isometric Exercises):ab,ti,kw OR (Isometric Exercise):ab,ti,kw OR (Exercise, Aerobic):ab,ti,kw OR (Aerobic Exercise):ab,ti,kw OR (Aerobic Exercises):ab,ti,kw

#3	#1 OR #2

#4	(Inflammatory Bowel Diseases):ab,ti,kw OR (Inflammatory Bowel Disease):ab,ti,kw OR (Bowel Diseases, Inflammatory):ab,ti,kw OR (inflammatory bowel disease):ab,ti,kw OR (Crohn's disease):ab,ti,kw OR (ulcerative colitis):ab,ti,kw

#5	(randomized controlled trial):ab,ti,kw OR (randomized):ab,ti,kw OR (placebo):ab,ti,kw OR (RCT):ab,ti,kw

#6	#3 AND #4 AND #5
