# Supplementary material for: Exercise-based and mind-body movement interventions for depressive symptoms in patients with inflammatory bowel disease: a systematic review and meta-analysis of randomized controlled trials
Source: Front Psychiatry. 2026 Jul 29;17:1887701. doi: 10.3389/fpsyt.2026.1887701 (PMC13461715; doi:10.3389/fpsyt.2026.1887701)
Supplement: Supplementary file 3 [file Supplementaryfile3.docx]

**Supplementary Table 3. Baseline depressive symptom scores and severity levels of included studies**

| **Study** | **Year** | **Depression scale** | **Intervention n** | **Intervention baseline score, mean (SD)** | **Intervention baseline severity** | **Control n** | **Control baseline score, mean (SD)** | **Control baseline severity** |
| --- | --- | --- | --- | --- | --- | --- | --- | --- |
| Zhou et al. (1) | 2025 | HADS-D | 25 | 5.76 (2.35) | Normal / non-clinical | 24 | 5.54 (2.45) | Normal / non-clinical |
| Zhou et al. (2) | 2025 | HADS-D | 26 | 5.35 (2.73) | Normal / non-clinical | 24 | 5.54 (2.45) | Normal / non-clinical |
| Tew et al. (1) | 2019 | HADS-D | 13 | 3.60 (3.10) | Normal / non-clinical | 11 | 5.20 (2.90) | Normal / non-clinical |
| Tew et al. (2) | 2019 | HADS-D | 12 | 3.80 (2.90) | Normal / non-clinical | 11 | 5.20 (2.90) | Normal / non-clinical |
| Gerbarg et al. | 2015 | BDI | 14 | 4.21 (4.15) | Minimal / non-clinical | 11 | 3.82 (2.86) | Minimal / non-clinical |
| Koch et al. | 2021 | HADS-D | 47 | 6.78 (3.84) | Normal / non-clinical | 50 | 7.00 (3.48) | Normal / non-clinical |
| Peerani et al. | 2022 | HADS-D | 49 | 4.98 (2.57) | Normal / non-clinical | 52 | 5.20 (2.85) | Normal / non-clinical |
| Cramer et al. | 2017 | HADS-D | 39 | 6.10 (3.90) | Normal / non-clinical | 38 | 6.50 (3.00) | Normal / non-clinical |
| Bauer et al. | 2024 | HADS-D | 19 | 6.60 (3.20) | Normal / non-clinical | 18 | 5.00 (2.80) | Normal / non-clinical |

**Note.** HADS-D = Hospital Anxiety and Depression Scale-Depression subscale; BDI = Beck Depression Inventory; SD = standard deviation. Severity interpretation was based on commonly used scale-specific cut-off criteria: HADS-D 0–7 = normal/non-clinical, 8–10 = borderline, and ≥11 = abnormal/clinically relevant depressive symptoms; BDI scores in the range shown were interpreted as minimal/non-clinical depressive symptoms. Mean baseline scores were used as approximate group-level indicators of depressive symptom severity.
